# The Cocktail Effect of BMP-2 and TGF-β1 Loaded in Visible Light-Cured Glycol Chitosan Hydrogels for the Enhancement of Bone Formation in a Rat Tibial Defect Model

**DOI:** 10.3390/md16100351

**Published:** 2018-09-25

**Authors:** Sun-Jung Yoon, Youngbum Yoo, Sang Eun Nam, Hoon Hyun, Deok-Won Lee, Sewook Um, So Yeon Kim, Sung Ok Hong, Dae Hyeok Yang, Heung Jae Chun

**Affiliations:** 1 Department of Orthopedic Surgery, Chonbuk National University Hospital, Jeonju 54907, Korea; sjyoon_kos@naver.com; 2 Department of Surgery, School of Medicine, The Konkuk University, Seoul 05030, Korea; 20010035@kuh.ac.kr (Y.Y.); 20090055@kuh.ac.kr (S.E.N.); 3 Department of Biomedical Sciences, Chonnam National University Medical School, Gwangju 61469, Korea; hhyun@chonnam.ac.kr; 4 Department of Oral & Maxillofacial Surgery, Kyung Hee University Dental Hospital at Gangdong, Kyung Hee University, Seoul 05278, Korea; verycutebear@hanmail.net; 5 Department of Veterinary Surgery, College of Veterinary Medicine, Seoul National University, Seoul 08826, Korea; umsewook@gmail.com; 6 Department of Dental Hygiene, College of Health Sciences, Cheongju University, Cheongju 28503, Korea; goodany00@naver.com; 7 Department of Dentistry, Catholic Kwandong University, School of Medicine, Medicine, International St. Mary’s Hospital, Incheon 22711, Korea; catherine.so.hong.sleepdoc@gmail.com; 8 Institute of Cell and Tissue Engineering, College of Medicine, The Catholic University of Korea, Seoul 06591, Korea; 9 Department of Biomedical Sciences, College of Medicine, The Catholic University of Korea, Seoul 06591, Korea; chunhj@catholic.ac.kr

**Keywords:** injectable hydrogel system, visible light-cured glycol chitosan, bone morphogenetic protein-2, transforming growth factor-beta1, bone formation, in vitro and in vivo

## Abstract

Bone tissue engineering scaffolds offer the merits of minimal invasion as well as localized and controlled biomolecule release to targeted sites. In this study, we prepared injectable hydrogel systems based on visible light-cured glycol chitosan (GC) hydrogels containing bone morphogenetic protein-2 (BMP-2) and/or transforming growth factor-beta1 (TGF-β1) as scaffolds for bone formation in vitro and in vivo. The hydrogels were characterized by storage modulus, scanning electron microscopy (SEM) and swelling ratio analyses. The developed hydrogel systems showed controlled releases of growth factors in a sustained manner for 30 days. In vitro and in vivo studies revealed that growth factor-loaded GC hydrogels have no cytotoxicity against MC3T3-E1 osteoblast cell line, improved mRNA expressions of alkaline phosphatase (ALP), type I collagen (COL 1) and osteocalcin (OCN), and increased bone volume (BV) and bone mineral density (BMD) in tibia defect sites. Moreover, GC hydrogel containing BMP-2 (10 ng) and TGF-β1 (10 ng) (GC/BMP-2/TGF-β1-10 ng) showed greater bone formation abilities than that containing BMP-2 (5 ng) and TGF-β1 (5 ng) (GC/BMP-2/TGF-β1-5 ng) in vitro and in vivo. Consequently, the injectable GC/BMP-2/TGF-β1-10 ng hydrogel may have clinical potential for dental or orthopedic applications.

## 1. Introduction

Various forms of biomaterials, such as hydrogels, films, sponges, fiber sheets, etc., have received extensive attention as tissue-engineered scaffolds due to their good biocompatibility and biodegradability as well as their simple processability [1]. Among biomaterials, hydrogels have some distinct advantages as tissue-engineered scaffolds because of a similar structure to extracellular matrix (ECM) and a porous structure that can support the adhesion and proliferation of cells by loading growth factors related to cell growth [2]. In particular, hydrogels allow for appropriate structures that facilitate the migration, adhesion, proliferation, and differentiation of osteoprogenitor cells into osteoblasts by the efficient delivery of nutrients and growth factors [3,4]. Such hydrogel systems prolong the half-life of growth factors, and maintain its stability and activation when incorporated [5]. As a hydrogel system, injectable hydrogel is of interest to scientists or clinicians engaged in tissue engineering applications due to their advantages, such as no biological damage of the irradiation, the controlled release of biomolecules by readily modulating cross-linking density, and the water solubility, biocompatibility and biodegradability of GC [6,7]. These remarkable characteristics give them clinical potential as bone tissue engineering scaffolds.

In addition to hydrogel scaffolds, the inclusion of several growth factors, such as bone morphogenetic protein-2 (BMP-2), growth and differentiation factor-5 (GDF-5) and transforming growth factor-beta1 (TGF-β1), have been found to be superior for bone repair [8,9,10]. Growth factor-loaded scaffolds have recently been used for bone formation and have included various combinations of growth factors. In this vein, we previously reported the enhancement of bone formation in vivo when using the combination of BMP-2 and GDF-5, which showed the cocktail effect of the growth factors on the improvement of bone formation in vitro and in vivo [8]. Also, the combination of BMP-2 and TGF-β1 can be a good candidate for the improvement of bone formation.

However, although TGF-β1 has excellent osteogenic activities such as the stimulation of matrix synthesis, dramatic effects on bone-forming osteoblasts and bone-resorbing osteoclasts, and inhibition of osteoclast precursors and bone resorption formation [9], little is known about the cocktail effect of BMP-2 and TGF-β1 on the improvement of bone formation [11]. Hence, many studies on the effects of combined BMP-2 and TGF-β1 for clinical uses in bone tissue engineering applications must be conducted.

In the present study, we prepared an injectable hydrogel system based on visible light-cured GC hydrogels containing BMP-2 and/or TGF-β1 and employed these systems as bone tissue regeneration scaffolds. The morphologies, storage and loss moduli, and swelling ratios of the developed hydrogels were characterized. The potential of BMP-2 (5 ng), TGF-β1 (5 ng), BMP-2/TGF-β1 (5 ng/5 ng) or BMP-2/TGF-β1 (10 ng/10 ng)-loaded GC hydrogels (GC/BMP-2, GC/TGF-β1, GC/BMP-2/TGF-β1-5 ng and GC/BMP-2/TGF-β1-10 ng) on the improvement of bone formation was evaluated using a rat tibial defect model.

## 2. Results

### 2.1. Storage Modulus

**Figure 1:**
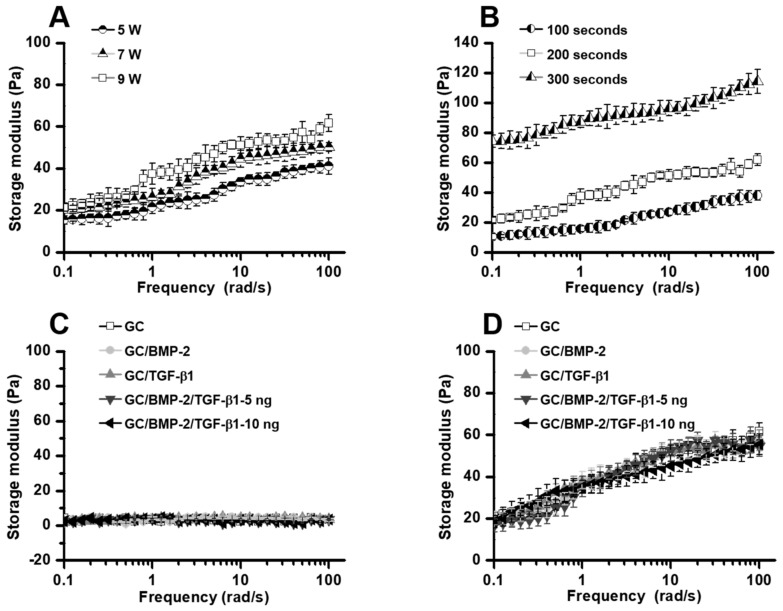
Storage moduli of GC irradiated at three kinds of (**A**) powers and (**B**) irradiation times. Storage moduli of GC, GC/BMP-2, GC/TGF-β1, GC/BMP-2/TGF-β1-5 ng and GC/BMP-2/TGF-β1-10 ng solutions (**C**) before and (**D**) after visible light irradiation for 200 s. The moduli were monitored from 0 Hz to 100 Hz. This experiment was carried out in trplicate.

Conjugation of glycidyl methacrylate (GM) to GC and its hydrogel formation was confirmed by our previous studies [6,7]. Rheometry was employed to investigate the storage moduli of hydrogels as a function of frequency (Figure 1). Figure 1A,B shows the storage moduli of GC hydrogel precursor solutions measured in various irradiation powers (5 W, 7 W and 9 W) and times (100 s, 200 s and 300 s). The results demonstrated that the storage moduli at 100 rad/s are power- and time-dependent, which had 41.1 Pa, 50.2 Pa and 61.7 Pa in the powers, and 37.9 Pa, 61.7 Pa and 114.2 Pa in the times, respectively. The hydrogel precursor solution irradiated with visible light for 300 s lost its injectable capacity due to increased elasticity; therefore, the irradiation conditions of 9 W and 200 s was chosen for the rapid procedure required for the animal test. The storage moduli of GC, GC/BMP-2, GC/TGF-β1, GC/BMP-2/TGF-β1-5 ng and GC/BMP-2/TGF-β1-10 ng hydrogel precursor solutions before and after visible light irradiation for 200 s with a power of 9 W are shown in Figure 1C,D. The hydrogel precursor solutions had a storage modulus below 5 Pa before the irradiation (Figure 1C), which increased to between 54 Pa and 62 Pa at 100 rad/s after irradiation with visible light (Figure 1D). These results demonstrated that visible light irradiation initiates a radical reaction that causes interconnections between the GM-conjugated GC (GM-GC) polymers, leading to hydrogel formation.

### 2.2. Morphologies

**Figure 2:**
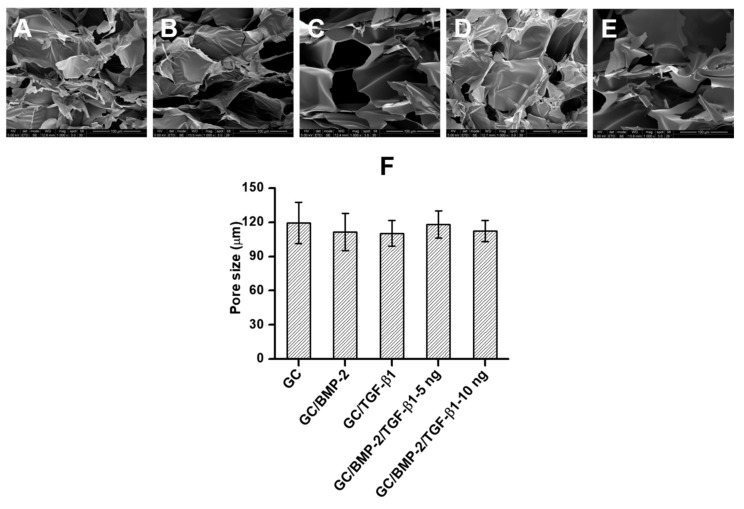
SEM images of freeze-dried (**A**) GC, (**B**) GC/BMP-2, (**C**) GC/TGF-β1, (**D**) GC/BMP-2/TGF-β1-5 ng and (**E**) GC/BMP-2/TGF-β1-10 ng hydrogels. The images were observed at a magnification of 1000×. (**F**) their pore sizes.

Scanning electron microscopy (SEM) was employed to observe the changes in the morphologies of freeze-dried GC, GC/BMP-2, GC/TGF-β1, GC/BMP-2/TGF-β1-5 ng and GC/BMP-2/TGF-β1-10 ng hydrogels. As shown in Figure 2A,E, porous structures were observed in the morphologies, along with interconnections among pores. The pore size and distribution were further investigated by Image-J and OriginPro 8, respectively. Marginal differences were observed in pore, irrespective of whether a growth factor was added. The results exhibited that GC, GC/BMP-2, GC/TGF-β1, GC/BMP-2/TGF-β1-5 ng and GC/BMP-2/TGF-β1-10 ng have 119.3 ± 18.2 µm, 111.5 ± 16.3 µm, 110.3 ± 11.2 µm, 118.2 ± 11.9 µm and 112.3 ± 9.3 µm of pore sizes, respectively (Figure 2F).

### 2.3. Swelling Ratio

**Figure 3:**
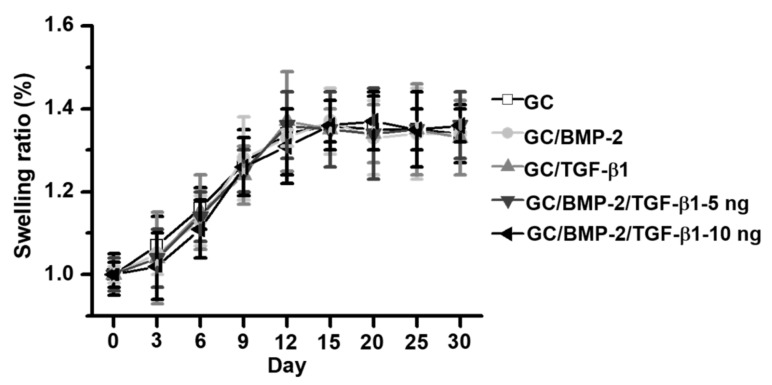
Swelling ratios of GC, GC/BMP-2, GC/TGF-β1, GC/BMP-2/TGF-β1-5 ng and GC/BMP-2/TGF-β1-10 ng hydrogels measured in PBS (pH 7.4) at predetermined time intervals (0, 3, 6, 9, 12, 15, 20, 25 and 30 days). This experiment was carried out in triplicate.

The swelling ratios of GC, GC/BMP-2, GC/TGF-β1, GC/BMP-2/TGF-β1-5 ng and GC/BMP-2/TGF-β1-10 ng hydrogels were measured for 30 days in phosphate buffered solution (PBS; pH 7.4) (Figure 3). A gradual increase was observed in the swelling ratios of the samples for 12 days, and they remained at an equilibrium state thereafter.

### 2.4. In Vitro Release Behavior of BMP-2 and/or TGF-β1

**Figure 4:**
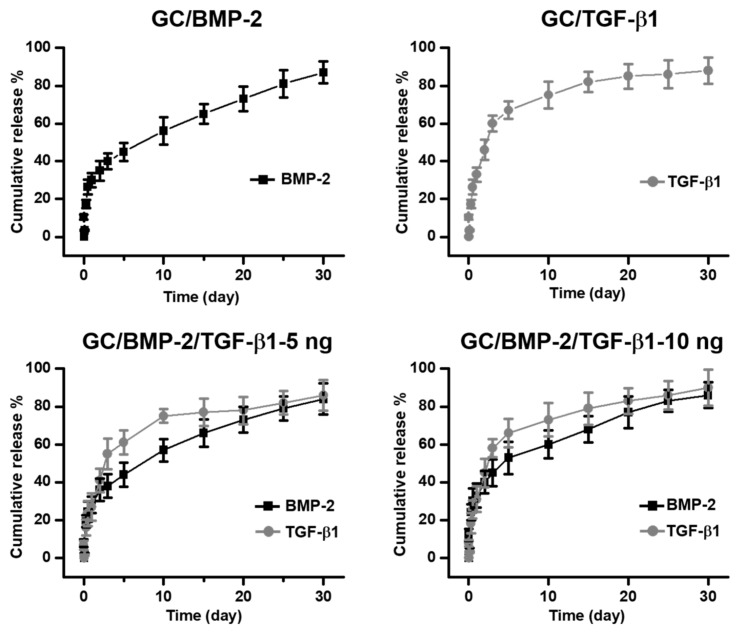
Release behaviors of BMP-2 and/or TGF-β1 from GC/BMP-2, GC/TGF-β1, GC/BMP-2/TGF-β1-5 ng and GC/BMP-2/TGF-β1-10 ng hydrogels. The measurements were carried out for 30 days. These experiments were carried out in triplicate.

The release behaviors of the growth factors from GC/BMP-2, GC/TGF-β1, GC/BMP-2/TGF-β1-5 ng and GC/BMP-2/TGF-β1-10 ng hydrogels are shown in Figure 4. The growth factor-loaded samples exhibited rapid releases for 24 h and controlled releases in a sustained manner thereafter. The cumulative release percentages for 30 days were 87%, 88%, 84%/86% and 86%/90% along with initial burst releases of 30%, 33%, 28%/27% and 33%/31%, respectively.

### 2.5. In Vitro Cell Proliferation of MC3T3-E1

**Figure 5:**
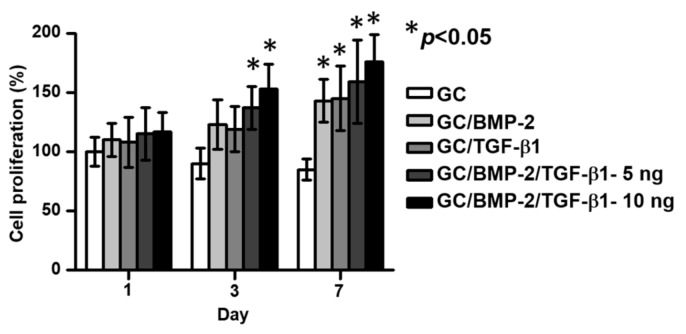
In vitro cell proliferation rates of MC3T3-E1 cultured on GC, GC/BMP-2, GC/TGF-β1, GC/BMP-2/TGF-β1-5 ng and GC/BMP-2/TGF-β1-10 ng hydrogels for 1, 3 and 7 days. The cell proliferation rate was measured using CCK-8 reagent kit. This experiment was carried out in triplicate (* *p* < 0.05). The control of “*” is GC.

Figure 5 shows the in vitro cell proliferations of MC3T3-E1 cultured in GC, GC/BMP-2, GC/TGF-β1, GC/BMP-2/TGF-β1-5 ng and GC/BMP-2/TGF-β1-10 ng hydrogels for 1, 3 and 7 days of culture. Compared to GC, gradual increases of MC3T3-E1 cell proliferation were observed over time in the growth factor-loaded hydrogels. On day 1, little difference in the cell proliferations was observed in the samples. On days 3 and 7, the growth factor-loaded samples had higher cell proliferation rates than that of GC. In addition, only GC/BMP-2/TGF-β1-5 ng and GC/BMP-2/TGF-β1-10 ng showed a statistical significance increase. Moreover, even though GC/BMP-2/TGF-β1-10 ng has no statistical significance in the cell proliferation rate compared to GC/BMP-2/TGF-β1-5 ng, it had the highest cell proliferation rate among growth factor-loaded GC hydrogels. The cell proliferation rates on day 7 were 2.1, 1.2, 1.2 and 1.1-fold higher than those of GC, GC/BMP-2, GC/TGF-β1 and GC/BMP-2/TGF-β1-5 ng, respectively.

### 2.6. mRNA Expression of Alkaline Phosphatase (ALP), Type I Collagen (COL 1) and Osteocalcin (OCN)

**Figure 6:**
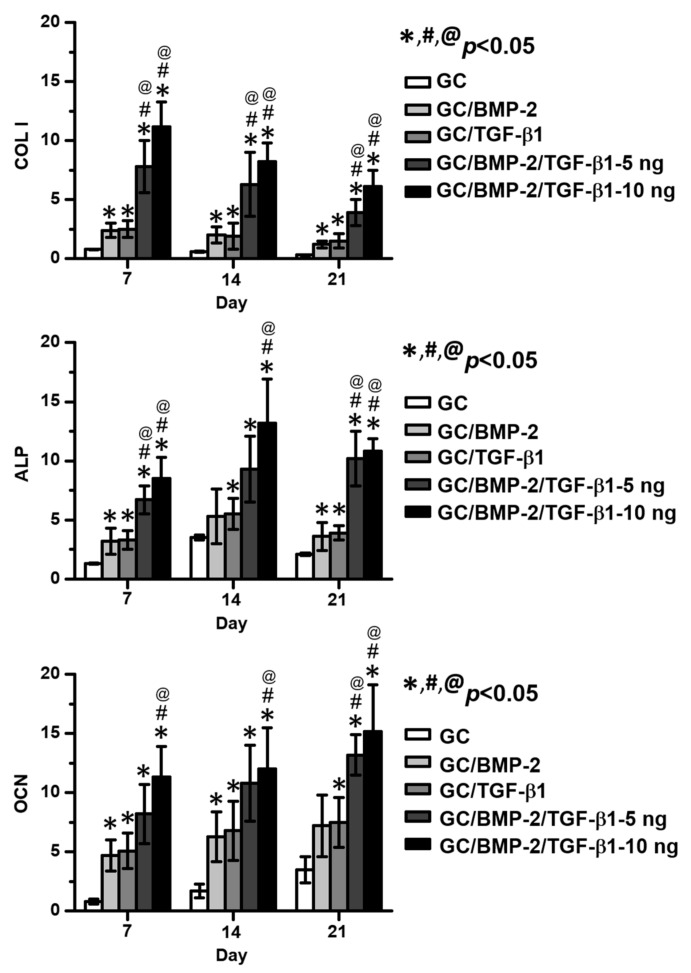
mRNA gene expression levels of COL1, ALP and OCN extracted from MC3T3-E1 cells cultured on GC, GC/BMP-2, GC/TGF-β1, GC/BMP-2/TGF-β1-5 ng and GC/BMP-2/TGF-β1-10 ng hydrogels for 7, 14 and 21 days. These experiments were carried out in triplicate (*^,#,@^ *p* < 0.05). The controls of “*”, “#” and “@” are GC, GC/BMP-2 and GC/TGF-β1, respectively.

Figure 6 shows COL 1, ALP and OCN mRNA expression in MC3T3-E1 cells cultured on GC, GC/BMP-2, GC/TGF-β1, GC/BMP-2/TGF-β1-5 ng and GC/BMP-2/TGF-β1-10 ng hydrogels for 7, 14 and 21 days. Over time, all cells exhibited gradual decreases of COL 1 and gradual increases of OCN. On the other hand, the expression of the ALP gene gradually increased for 14 days and decreased thereafter. Gene expression in the growth factor-loaded GC hydrogels was greater than that in GC. The average gene expressions of the cells cultured on GC/BMP-2/TGF-β1-10 ng were greater than those on GC/BMP-2/TGF-β1-5 ng. In particular, GC/BMP-2/TGF-β1-10 ng exhibited the greatest gene expression among the growth factor-loaded GC hydrogels.

### 2.7. Radiographical and Micro Computed Tomography (CT) Assays

**Figure 7:**
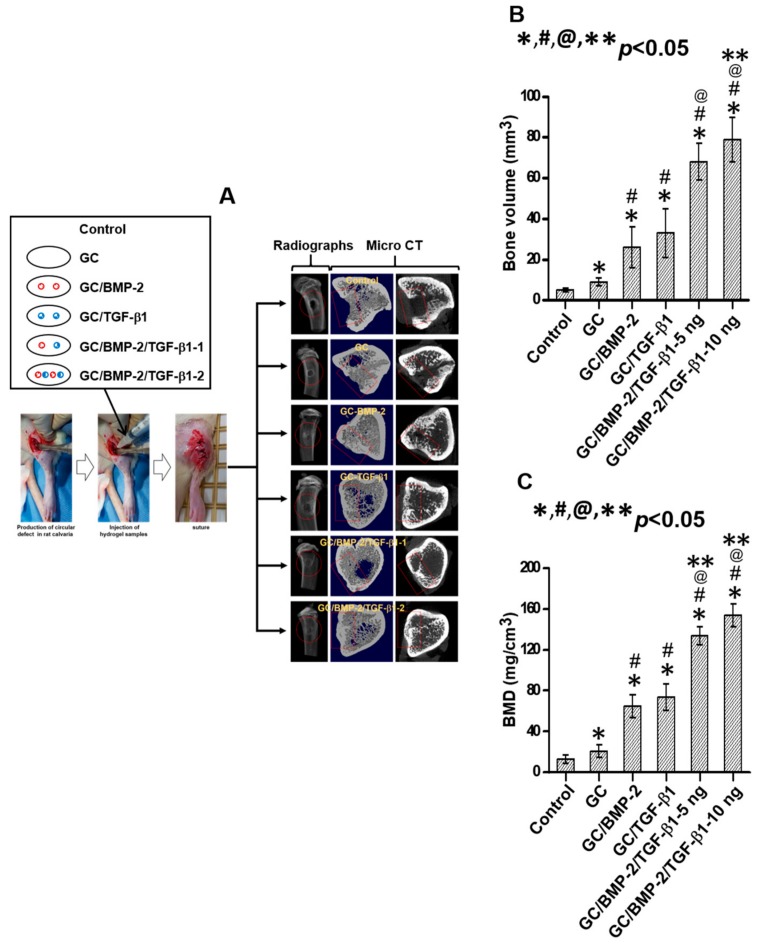
(**A**) Experimental procedures of animal test, and the results of radiographs and micro CT images, and (**B**) BVs and (**C**) BMDs of control tibial defect, and tibial defects treated with GC, GC/BMP-2, GC/TGF-β1, GC/BMP-2/TGF-β1-5 ng and GC/BMP-2/TGF-β1-10 ng hydrogels for 4 weeks of implantation. This experiment was carried out five times (*^,#,@,^** *p* < 0.05). The controls of “*”, “#”, “@” and “**” are control, GC, GC/BMP-2 and GC/TGF-β1, respectively.

Figure 7 shows experimental procedures, the radiographs and micro CT images, and bone volumes (BVs) of the tibia defects in control, and in rats implanted with GC, GC/BMP-2, GC/TGF-β1, GC/BMP-2/TGF-β1-5 ng and GC/BMP-2/TGF-β1-10 ng for 4 weeks. Mineralized bone was remarkably formed in the tibia defects treated with growth factor-loaded GC hydrogels (Figure 7A). To further investigate the bone formation, the BVs and BMDs in the defected sites were measured (Figure 7B,C). Sites implanted with GC and growth factor-loaded GC hydrogels had significantly higher BVs and BMDs than those of control (untreated). In addition, the BV on GC/TGF-β1 was slightly larger than that on GC/BMP-2 even if no statistical significance is between the two hydrogels. Injection of two kinds of GC/BMP-2/TGF-β1 hydrogels resulted in noticeable bone formation at the defected site, which can be attributed to the combined effect of BMP-2 and TGF-β1. Moreover, GC/BMP-2/TGF-β1-10 ng implantation formed a larger amount of bone than GC/BMP-2/TGF-β1-5 ng although no statistical significance is between the growth factor-loaded hydrogels. The BV of GC/BMP-2/TGF-β1-10 ng was 15.8, 8.8, 3.0, 2.4 and 1.2-fold larger than those of GC, GC/BMP-2, GC/TGF-β1 and GC/BMP-2/TGF-β1-5 ng, respectively (Figure 7B). The BMD was 11.9, 7.3, 2.4, 2.1 and 1.2-fold larger than those of GC, GC/BMP-2, GC/TGF-β1 and GC/BMP-2/TGF-β1-5 ng, respectively (Figure 7C).

### 2.8. Histological Evaluations

**Figure 8:**
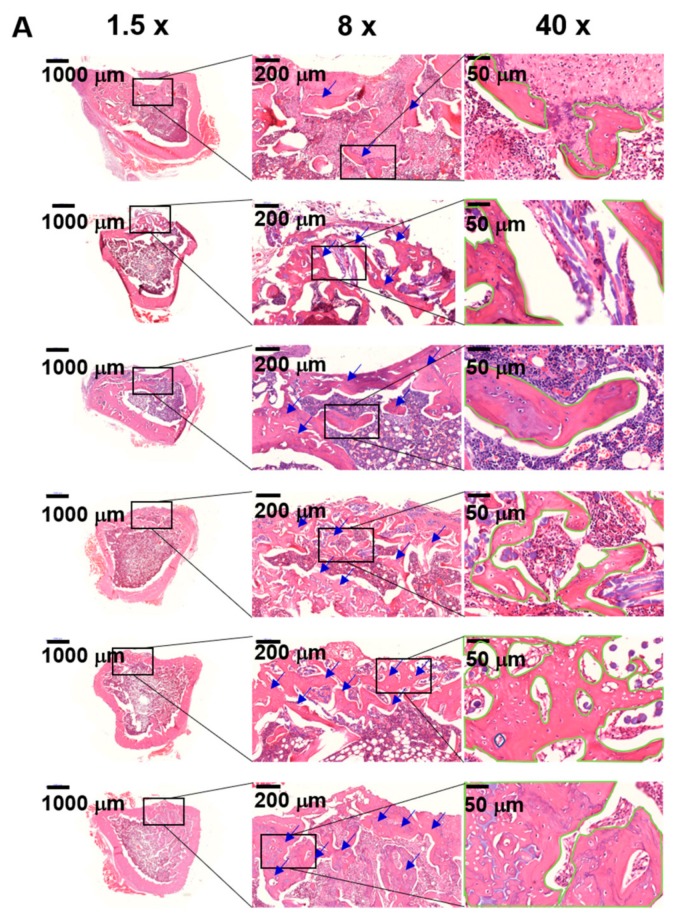

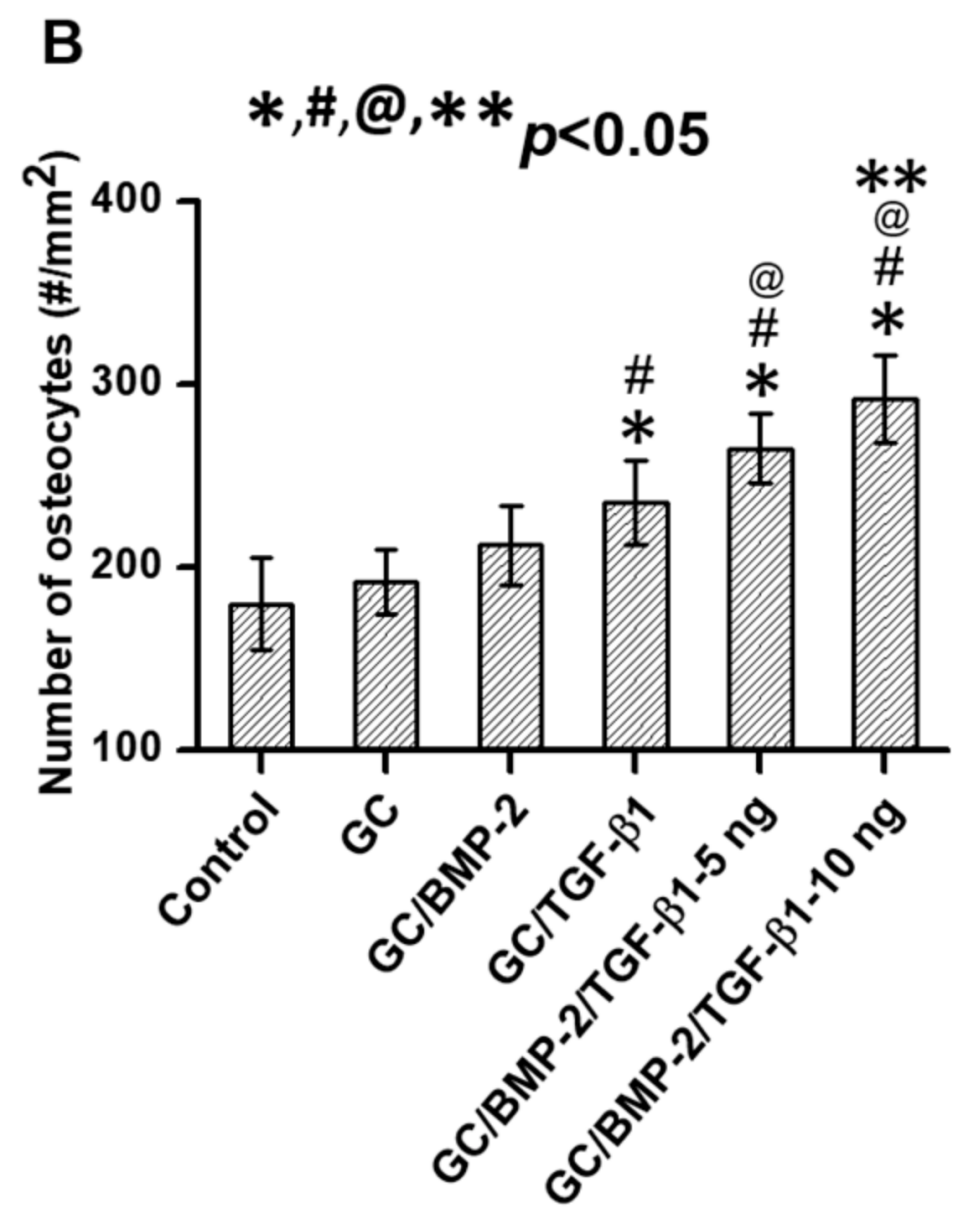
H&E-stained images of control tibial defect, and tibial defects treated with GC, GC/BMP-2, GC/TGF-β1, GC/BMP-2/TGF-β1-5 ng and GC/BMP-2/TGF-β1-10 ng hydrogels for 4 weeks of implantation observed at magnifications of (**A**) 1.5× and 8×, and 40×. (**B**) Number of osteocytes observed in the defect sites. In H&E-stained images of 8× and 40×, the blue arrows and the green lines indicate osteocyte in lacunae and newly formed bone, respectively. The scale bars at 1.5×, 8×, and 40× indicate 1000 µm, 200 µm and 50 µm, respectively. This experiment was carried out five times (*^,#,@,^** *p* < 0.05), and the data shown in Figure (**A**) is a representative of H&E-stained images. The controls of “*”, “#”, “@” and “**” are control, GC, GC/BMP-2 and GC/TGF-β1, respectively.

**Figure 9:**
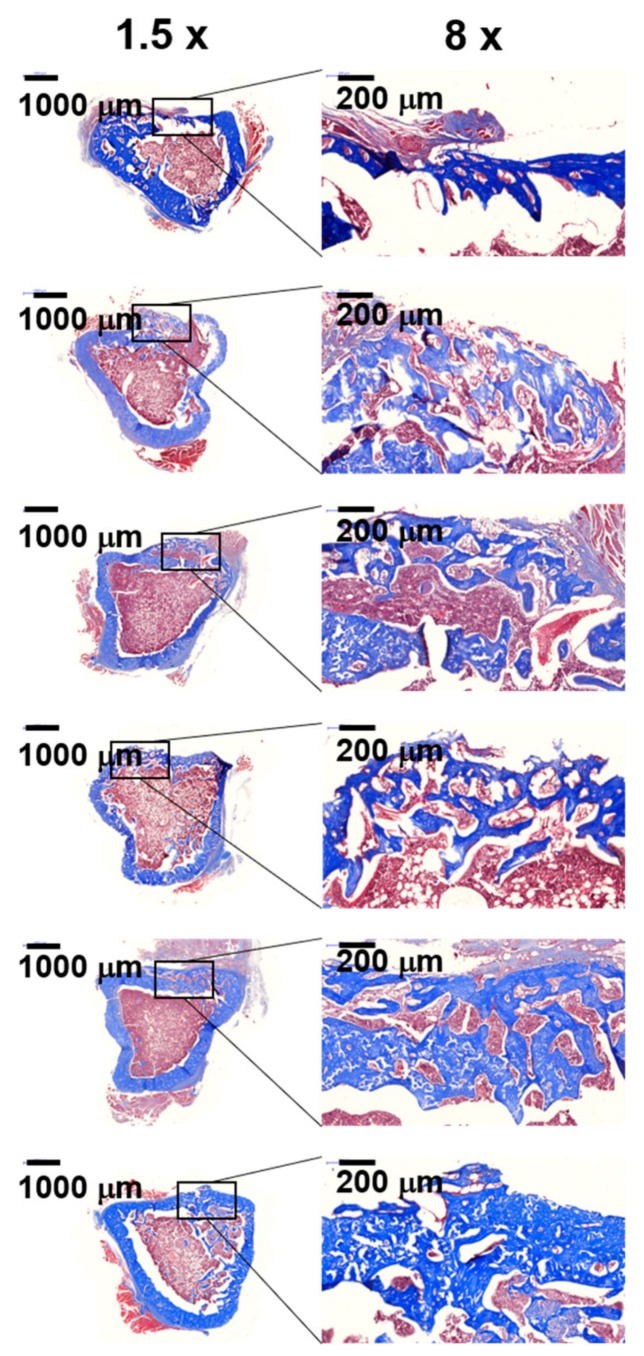
MT-stained images of control tibial defect, and tibial defects treated with GC, GC/BMP-2, GC/TGF-β1, GC/BMP-2/TGF-β1-5 ng and GC/BMP-2/TGF-β1-10 ng hydrogels for 4 weeks of implantation observed at magnifications of 1.5× and 8×. In H&E-stained images of 1.5× and 8×, the blue colored areas indicate the formed collagen matrix. The scale bars at 1.5× and 8× indicate 1000 µm and 200 µm, respectively. This experiment was carried out five times, and the data shown in Figure 9 is a representative of MT-stained images.

The histological evaluations of the defected sites treated with GC, GC/BMP-2, GC/TGF-β1, GC/BMP-2/TGF-β1-5 ng and GC/BMP-2/TGF-β1-10 ng hydrogels were compared with those of control and site treated with GC, as shown in Figure 8 and Figure 9. In hematoxylin & eosin (H&E) stained images, lots of osteocytes occupied in lacunae were observed in the defected sites treated with growth factor-loaded GC hydrogels, as compared with those of control and treated with GC (Figure 8A). In addition to the stained images, the result of Figure 8B exhibited that two kinds of GC/BMP-2/TGF-β1 hydrogels have a larger number of osteocytes than GC, GC/BMP-2 and GC/TGF-β1. Moreover, even though there is no statistical significance between GC/BMP-2/TGF-β1-5 ng and GC/BMP-2/TGF-β1-10 ng, the latter had larger number of osteocytes than the former. This demonstrated that partially mineralized bone was observed in the defected sites untreated (control) and treated with GC, while a great amount of mineralized bone formed in sites in which growth factor-loaded GC hydrogels had been implanted. Collagen formed in the defected sites was observed by Masson’s trichrome (MT) stain (Figure 9). The MT-stained images revealed that a remarkable amount of collagen formed in the defected sites treated with growth factor-loaded GC hydrogels, as compared to collagen formed in the defected sites of control and rats treated with GC. Moreover, treatment with GC/BMP-2/TGF-β1-10 ng resulted in improved mineralized bone formation.

## 3. Discussion

In this study, we prepared injectable hydrogel systems by photocuring water-soluble GC with riboflavin as a visible light initiator, allowing for the controlled release of BMP-2 and/or TGF-β1. In addition, we investigated the feasibility of growth factor-loaded GC hydrogels leading to improved bone formation using a rat model with a tibia defect. As we previously reported, the conversion of hydrogel precursor solution into hydrogel was confirmed by measuring the storage modulus as characterized by rheometry [6,7]. The precursor solution had a storage modulus below 5 Pa, which increased after visible light irradiation because a hydrogel was produced by the formation of three-dimensional networks between the GM-GC chains (Figure 1).

In addition to an increased storage modulus, the hydrogels were found to have an interconnected, porous structure and to have swelled in the aqueous solution (Figure 2 and Figure 3). Previous reports discussed hydrogel characteristics in terms of the release behaviors of biomolecules such as drugs and growth factors. These releases can be primarily ascribed to swelling of the hydrogel and diffusion of biomolecules [12,13,14,15,16]. Growth factors that are pre-loaded in the porous structure are released when water diffuses into the hydrogels, causing swelling. Consequently, the growth factors close to the hydrogel surface are rapidly released, followed by controlled and sustained release of the growth factors in the hydrogel core, which corresponds well with the results obtained in this investigation (Figure 4).

Cell and tissue responses to biomaterials are essential considerations for the design and preparation of tissue engineering scaffolds in various medical fields. Therefore, the in vitro cytotoxicity test is usually employed to evaluate these responses [17]. In our previous study, we investigated the cytocompatibility of a visible light-cured GC hydrogel with the L-929 cell line [6,7]. This hydrogel was also found to be cytocompatible with the MC3T3-E1 osteoblast cell line (Figure 5). Moreover, the growth factor-loaded hydrogels resulted in an improved MC3T3-E1 cell proliferation (Figure 5).

As mentioned above, polymeric hydrogels with three-dimensional networks generally have a porous structure, leading to various pore sizes caused by variations in crosslinking density [18]. In visible light-cured hydrogel systems, pore size can be easily controlled by how long the hydrogels are irradiated, because the degree of crosslinking density depends on the length of irradiation time [19]. In bone tissue engineering, scaffolds with pore sizes ranging from 100 to 400 µm are known to improve osteoconduction, while vascularization occurs well in scaffolds with pore sizes greater than 300 µm [20,21]. However, pores smaller than 100 µm may induce development of endochondral during bone formation [20,21]. In this study, the GC-based hydrogels, GC, GC/BMP-2, GC/TGF-β1, GC/BMP-2/TGF-β1-5 ng, and GC/BMP-2/TGF-β1-10 ng, were found to have pore sizes between 80 and 100 µm when irradiated for 200 s (Figure 2). Considering the appropriate pore sizes for improving osteogenic activity, the obtained pore size should provide the proper circumstances for proliferation and differentiation of bone-related cells (Figure 6). This was confirmed by micro CT and histological analyses (Figure 7, Figure 8 and Figure 9).

Micro CT analysis revealed that the GC-based hydrogels result in a remarkable improvement in bone formation. In addition, incorporation of BMP-2 and/or TGF-β1 in the GC hydrogels accelerates bone formation by promoting to proper bone remodeling (Figure 7). These results can be attributed to the cocktail effect of BMP-2 and TGF-β1. Mesenchymal stem cells from the bone marrow migrate into the growth factor-loaded GC hydrogels implanted at the defected site, adhere to cationic GC chains, and differentiate into preosteoblasts, osteoblasts, and osteocytes. Consequently, the hydrogels loaded with these growth factors may accelerate osteogenic activity. Furthermore, an appropriate concentration of BMP-2 and TGF-β1 can improve osteogenic activity. The results of micro CT were further proved by histological evaluations. Active osteocytes and lots of collagen synthesis were clearly observed in H&E and MT-stained images of samples treated with GC/BMP-2/TGF-β1-10 ng (Figure 8 and Figure 9). We therefore conclude that GC/BMP-2/TGF-β1-10 ng may have potential for clinical use in the orthopedic and dental fields.

## 4. Materials and Methods

### 4.1. Materials

Glycol chitosan (GC) and glycidyl methacrylate (GM) were purchased from Sigma-Aldrich (St. Louis, MO, USA). Bone morphogenetic protein-2 (BMP-2) and transforming growth factor-beta1 (TGF-β1) were purchased from Thermo Fisher Scientific (Waltham, MA, USA). 4-(4,6-Dimethoxy-1,3,5-triazin-2-yl)-4-methylmorpholinium chloride (DMT-MM) was obtained from Wako Pure Chemical Industries (Osaka, Japan). Dialysis tubes (Spectrum Laboratories Inc., Rancho Dominguez, CA, USA) were used for purification. The MC3T3-E1 cell line was purchased from the American Type Culture Collection (ATCC; Manassas, VA, USA). α-Minimum Eagle’s medium, fetal bovine serum (FBS), and penicillin and streptomycin (PS) were supplied by Life Technologies (Grand Island, NY, USA). All chemicals were used as received.

### 4.2. Preparation of Growth Factor-Loaded GC Hydrogels

Conjugation of GM to GC was performed as reported previously [6,7]. In brief, GM liquid (0.05 mmol, 7 mg) was dropped into an aqueous GC solution (0.003 mmol, 1.5 g) adjusted to pH 9 in distilled water (50 mL) and continuously stirred at room temperature for 2 days. The reactant was dialyzed using a cellulose membrane tube (cut-off: 20 kDa) in water for 3 days and lyophilized at 90 °C for 7 days. BMP-2 and/or TGF-β1 (10, 10, 5/5, and 10/10 ng) were added to a GM-GC solution (1 w/v%) in PBS (1 mL) and homogeneously mixed. Two concentrations of BMP-2 (5 ng) and TGF-β1 (5 ng) were selected for this study because the specific concentrations are known to improve bone formation [22,23]. In addition, two-fold concentrations of BMP-2 (10 ng) and TGF-β1 (10 ng) were used to examine concentration dependency on the improvement of bone formation in vitro and in vivo. Prior to the visible light irradiation of the hydrogel precursor solutions, each growth factor-loaded GC hydrogel was added to the solutions and photocured using a blue light (430–485 nm 2100 M_W_/cm^2^, light-emitting diode (LED) curing light, Forshan Keyuan Medical Equipment Co., Ltd.; Foshan, China) for 200 s.

### 4.3. Storage Modulus

Rheological properties on all hydrogel samples were measured using the AR 2000 EX rheometer (TA instrument, New Castle, DE, USA) set with a cone and plate geometry of 4 cm diameter and 1° cone angel as a function of frequency. A fixed sample volume was placed on the rotational cone and plate and measured at 37 °C at a range of 0 to 100 Hz.

### 4.4. Scanning Electron Microscopy

Freeze-dried hydrogel samples were fixed onto metal mounts using carbon tape and then gold-coated using an ion sputter-coater (Eiko IB-3, Eiko Engineering Co. Ltd., Tokyo, Japan). The morphologies were observed by SEM (Inspect F; FEI, Hillsboro, Oregon, USA) at 30.0 kV with a magnification of 1000×. Image-J (NIH, Bethesda, MD, USA) and OriginPro 8 (OriginLab Corp., Northampton, MA, USA) programs were employed to investigate the pore size and distribution, respectively.

### 4.5. Swelling Ratio

At predetermined time intervals (0, 3, 6, 9, 12, 15, 20, 25 and 30 days), each hydrogel sample was extracted from PBS (pH 4) and washed with distilled water three times. After removing water on the surface, the samples were weighted. The swelling ratio was calculated by the ration of the swollen weight to the initial weight of the hydrogels.

### 4.6. Release Behavior of BMP-2 and/or TGF-β1

Growth factor-loaded GC hydrogels were added to glass vials containing PBS (3 mL; pH 7.4) and incubated at 37 °C for 30 days under continuous agitation at 100 rpm. At predetermined time intervals (1, 3, 7, and 12 h, and 1, 2, 3, 5, 10, 15, 20, 25, and 30 days), 2 mL of PBS was collected from each sample, and the same volume of PBS was added. Two types of enzyme-linked immunosorbent assay (ELISA) kits (Bio-Rad, Hercules, CA, USA for BMP-2 and eBioscience, San Diego, CA, USA for TGF-β1) were used to analyze the release behavior of the growth factors [24,25]. Prior to the analyses, the supernatants were stored at −20 °C. Standard calibration curves were prepared by measuring various known concentrations of BMP-2 and TGF-β1 solution (R_2_: 0.998 for BMP-2; 0.995 for TGF-β1). Cumulative release percentages of BMP-2 and/or TGF-β1 were calculated in comparison with each of the standard calibration curve.

### 4.7. Cell Proliferation of MC3T3-E1

Prior to the cell proliferation assay, GC hydrogel precursor solution was sterilized for 15 min in an autoclave set at 121 °C. After mixing it with growth factors, the mixtures (150 µL) were placed in 96-well plates and photocured with blue light. The MC3T3-E1 cell line (1 × 10^4^ cells/well) was seeded in each well containing the hydrogel samples and cultured with α-minimum Eagle’s medium containing 10% FBS and 1% PS at 37 °C in a humidified 5% CO_2_ environment. At predetermined time intervals (1, 3, and 7 days), the cultured cells were treated with cell counting kit-8 (CCK-8) reagent (100 µL) and incubated for 2 h. The supernatants collected from each well were measured using a microplate reader (SpectraMax^®^ i3; Molecular Devices, Sunnyvale, CA, USA) at 450 nm.

### 4.8. mRNA Expression Assays

The mRNA expression levels of COL 1, ALP, and OCN were analyzed to evaluate the early, middle, and final differentiations of the MC3T3-E1 cells (initial seeding amount: 5 × 10^5^ cells/well) cultured on GC, GC/BMP-2, GC/TGF-β1, GC/BMP-2/TGF-β1-5 ng, and GC/BMP-2/TGF-β1-10 ng hydrogels in 6-well plates for 7, 14, and 21 days, respectively. The cells were cultured with osteogenic medium supplemented with 10% FBS, 1% PS, 10 mM β-glycero phosphate disodium salt hydrate, 300 µM ascorbic acid, and 0.1 µM dexamethasone. At each predetermined time interval, the total RNA of the cells cultured on each hydrogel sample was isolated using a RNeasy Plus Mini Kit (Qiagen, Hilden, Germany). The extracted RNA (1 µg) was transcribed into cDNA using an AccuPower CycleScript RT Premix (Bioneer, Daejeon, Korea). The real-time polymerase chain reaction (qPCR) amplifications were conducted using an AccuPower PCR PreMix (Bioneer, Daejeon, Korea) and were detected using an iQ SYBR Green Supermix (Bio-Rad, Hercules, CA, USA). The following oligonucleotide primers were used: COL 1 (accession number: XM005257059.3), sense 5′-GTGAGACAGGCGAACAAG-3′, antisense 5′-CAGGAGAACCAGGAGGAC-3′; ALP (accession number: XM017001582.1), sense 5′-CCAGCAGGTTTCTCTCTTGG-3′, antisense 5′-GACTGTAGGGACGATTGGA-3′; OCN (accession number: NM172209.2), sense 5-ATGAGGACCCTCTCTCTGCT-3, antisense 5′-CGTAGATGCGTTTGTAGGC-3′; and glyceraldehyde 3-phosphate dehydrogenase (GAPDH) (accession number: nm001289746.1), sense 5′-ACTTTGTCAAGCTCATTTCC-3′, antisense 5′-TGCAGCGAACTTTATTGATG-3′ [8]. The initial denaturation was conducted for 10 min at 95 °C, and the qPCR amplifications were then carried out with a cycle of 10 s at 95 °C, 30 s at 57–62 °C (*COL 1*: 60 °C, *ALP*: 61 °C, *OCN*: 57 °C), and 30 s at 72 °C for 45 cycles.

### 4.9. In Vivo Animal Test

The animal test was approved by the Institutional Animal Care and Use Committee (IACUC) of the Kyung Hee University Hospital at Gangdong (No. KHNMC AP 2016-008). Tested animals (30 male white wistar rats; 4~8 weeks; 180~220 g molecular weight) were anesthetized with Ketara^®^ (50 mg/kg) and Rompun^®^ (5 mg/kg). After shaving their thighs, 3 mm bore holes were first produced in the cortical bone to facilitate hydrogel injection using syringes. Each hydrogel sample was injected into the defected hole. After suturing the periosteum and fascia using Ethicon Vicryl^®^, skin was closed using Ethicon Ethilon^®^. After 4 weeks, the animals were euthanized, and the implanted sites were harvested for micro CT and histological evaluations. This experiment was carried out on each hydrogel samples as well as control (untreated) five times.

### 4.10. Micro CT Analysis

The BVs and BMDs formed in the implanted sites were evaluated by micro CT analysis (Skyscan 1076, Kontich, Belgium). The images were scanned with an X-ray source voltage of 100 kV and source current of 100 µA. During acquiring images at 0.6° angular increments over 360°, each frame was exposed for 460 ms. The scanned images were analyzed by Skyscan^TM^ CT-analyzer software. For calibration of the acquired image values in Hounsfield units, the CT numbers of air (−1000 HU) and water (0 HU) were used [26].

### 4.11. Histological Evaluation

After decalcification in 10% ethylenediaminetetraacetic acid (pH 7.4), the excised wound sites were fixed in 10% neutral formaldehyde solution. The fixed tissues were embedded in paraffin and sectioned at 3 µm. Sections were evaluated histologically by H&E and MT staining. The stained slides were observed by light microscopy (IX71 inverted microscope, Olympus, Tokyo, Japan) [27,28]. In H&E-stained images at a magnification of 40×, the number of osteocytes in lacunae on each sample was estimated by calculating the number in approximately 1 mm^2^ [29]. The number of osteocytes in lacunae per bone area was expressed as N.Ot/B.Ar.

### 4.12. Statistical Analysis

All quantitative data were expressed as the mean ± standard deviation. One-way analysis of variance (ANOVA) was carried out using SPSS software (SPSS Inc., Chicago, IL, USA). *^,#,@,^** *p* value < 0.05 were considered statistically significant. The number of each sample for in vivo animal testing was calculated by MedCalc Statistical Software (MedCalc Software bvba, Ostend, Belgium) using α (*p* = 0.05) and power (1β = 0.8).

## 5. Conclusions

In this study, we prepared an injectable system of GC hydrogels cured by visible light and containing BMP-2 and/or TGF-β1 as bone tissue engineering scaffolds for the improvement of bone formation in vivo. Hydrogels with appropriate pore sizes and storage moduli exhibited controlled and sustained release of the growth factors and improved the cell proliferation and differentiation of MC3T3-E1 osteoblasts in vitro. Furthermore, the hydrogels accelerated bone formation in the tibia defect sites of rat models. In addition, GC hydrogels loaded with BMP-2 and/or TGF-β1 showed superior bone formation abilities at the defect site. Consequently, we propose that GC/BMP-2/TGF-β1-10 ng may have potential for clinical use in the orthopedic and dental fields.
